# Angiopoietin-like 4 induces a β-catenin-mediated upregulation of ID3 in fibroblasts to reduce scar collagen expression

**DOI:** 10.1038/s41598-017-05869-x

**Published:** 2017-07-24

**Authors:** Ziqiang Teo, Jeremy Soon Kiat Chan, Han Chung Chong, Ming Keat Sng, Chee Chong Choo, Glendon Zhi Ming Phua, Daniel Jin Rong Teo, Pengcheng Zhu, Cleo Choong, Marcus Thien Chong Wong, Nguan Soon Tan

**Affiliations:** 1 School of Biological Sciences, Nanyang Technological University, 60 Nanyang Drive, Singapore, 637551 Singapore; 2 School of Materials Science and Engineering, Nanyang Technological University, Nanyang Avenue, Singapore, 639798 Singapore; 3 Lee Kong Chian School of Medicine, Experimental Medicine Building, 59 Nanyang Drive, Singapore, 636921 Singapore; 4 Tan Tock Seng Hospital, 11 Jalan Tan Tock Seng, Singapore, 308433 Singapore; 5 Institute of Molecular and Cell Biology, 61 Biopolis Drive, Proteos, A*STAR Singapore, 138673 Singapore; 6 KK Research Centre, KK Women’s and Children’s Hospital, 100 Bukit Timah Road, Singapore, 229899 Singapore; 7 Denova Sciences Pte. Ltd., Singapore, Singapore

## Abstract

In adult skin wounds, collagen expression rapidly re-establishes the skin barrier, although the resultant scar is aesthetically and functionally inferior to unwounded tissue. Although TGFβ signaling and fibroblasts are known to be responsible for scar-associated collagen production, there are currently no prophylactic treatments for scar management. Fibroblasts in crosstalk with wound keratinocytes orchestrate collagen expression, although the precise paracrine pathways involved remain poorly understood. Herein, we showed that the matricellular protein, angiopoietin-like 4 (ANGPTL4), accelerated wound closure and reduced collagen expression in diabetic and ANGPTL4-knockout mice. Similar observations were made in wild-type rat wounds. Using human fibroblasts as a preclinical model for mechanistic studies, we systematically elucidated that ANGPTL4 binds to cadherin-11, releasing membrane-bound β-catenin which translocate to the nucleus and transcriptionally upregulate the expression of Inhibitor of DNA-binding/differentiation protein 3 (ID3). ID3 interacts with scleraxis, a basic helix-loop-helix transcription factor, to inhibit scar-associated collagen types 1α2 and 3α1 production by fibroblasts. We also showed ANGPTL4 interaction with cadherin-11 in human scar tissue. Our findings highlight a central role for matricellular proteins such as ANGPTL4 in the attenuation of collagen expression and may have a broader implication for other fibrotic pathologies.

## Introduction

Cutaneous wound repair is a vital process that restores skin integrity after injury. The wound healing process consists of three integrated and overlapping phases: inflammation, re-epithelialization, and tissue remodeling. The numerous biological processes that occur during wound healing are orchestrated by a variety of cell types and their dynamic interactions within the wound microenvironment^1, 2^. Localized collagen expression rapidly replaces the missing tissue to allow successful resolution of local injury. In adult skin, this results in scar tissue that has inferior skin function, biomechanical properties, and aesthetic quality which significantly contributes to physical and psychological morbidity^3, 4^. Fibroblasts are a major component of the skin dermis and play crucial roles in the laying down and remodeling of extracellular matrix components during wounding. Numerous studies have demonstrated the importance of collagen expression and the resulting organization of collagen fibrils during scar formation. Scar tissues consistently have a higher level of collagen type 1α2 (COL1A2) and 3α1 (COL3A1) compared with scar-free wounds. The complex process of scar formation involves molecular controls that are still largely unknown, hence there are currently no registered pharmaceuticals for the prophylactic improvement of scarring and no single therapy is accepted as the universal standard^5, 6^. Scar improvement and management thus remains an area of clear medical need^7, 8^.

Matricellular proteins are secreted into the extracellular environment but do not play a primary structural role. Rather, they modulate several regulatory networks that are important for cell-matrix and cell-cell communication^9^. Importantly, these molecular networks may create many opportunities for the compensatory adjustments that are necessary for wound repair. However, existing scar management and therapeutic strategies have overlooked the central role of matricellular proteins in orchestrating cellular events during wound healing and the scarring response. The matricellular protein angiopoietin-like 4 (ANGPTL4) was recently reported to play important roles during wound repair^10^. Full-length ANGPTL4 undergoes proteolytic cleavage, releasing the N-terminal coiled-coil domain (nANGPTL4) and the C-terminal fibrinogen-like domain (cANGPTL4). While nANGPTL4 has well-established roles in peripheral triglyceride metabolism, cANGPTL4 regulates diverse cellular functions to facilitate wound repair^11–14^.

During the healing of a full-thickness excisional wound in mouse skin, ANGPTL4 mRNA peaked at day 3–5 post wounding. Immunofluorescence staining and western blot analysis showed that the expression of cANGPTL4 protein peaked at day 5 in the wound epidermis and remained elevated until day 15 upon wound closure^12^. cANGPTL4 interacts with integrins β1 and β5 in keratinocytes to stimulate proliferation and migration^11^. cANGPTL4 also interacts with vitronectin and fibronectin in the wound bed, delaying their proteolytic degradation by metalloproteinases. This interaction does not interfere with integrin-matrix protein recognition and directly affects cell-matrix communication by altering the availability of intact matrix proteins^11, 12^. The cANGPTL4:integrin signaling axis in keratinocytes regulates inducible nitric oxide synthase (iNOS) expression to increase nitric oxide (NO) levels within the wound bed, thereby improving wound angiogenesis to accelerate wound repair^14^. The expression of ANGPTL4 was transcriptionally upregulated by the nuclear receptor peroxisome proliferator activated receptor β/δ (PPARβ/δ) as determined by immunofluorescence staining and qPCR of laser-capture microdissected wound epidermis^12^. In poor healing diabetic wounds, the expression of cANGPTL4 remained low throughout the healing period^14^. Histomorphormetric analysis of ANGPTL4-deficient and wild-type mice showed that the re-epithelialization and remodeling phases of wound healing were affected by the absence of ANGPTL4 in mouse skin^11–13^. Consistently, wounds in ANGPTL4-deficient mice exhibited delayed healing, increased inflammation, and impaired wound-related angiogenesis^11, 12^. However, the role of ANGPTL4 in fibroblasts and its effects on scar-associated collagen production is unknown.

In this study, we demonstrated that cANGPTL4 decreases scar-associated collagen (COL1A2 and COL3A1) expression in wound fibroblasts. We systemically elucidated the mechanism which involves β-catenin-mediated upregulation of ID3 activated by cANGPTL4 without antagonizing the canonical TGFβ-Smad3 signaling in fibroblasts.

## Results

### cANGPTL4 accelerates wound healing, modifies wound collagen expression and improves the biomechanical quality of healed skin

**Figure 1:**
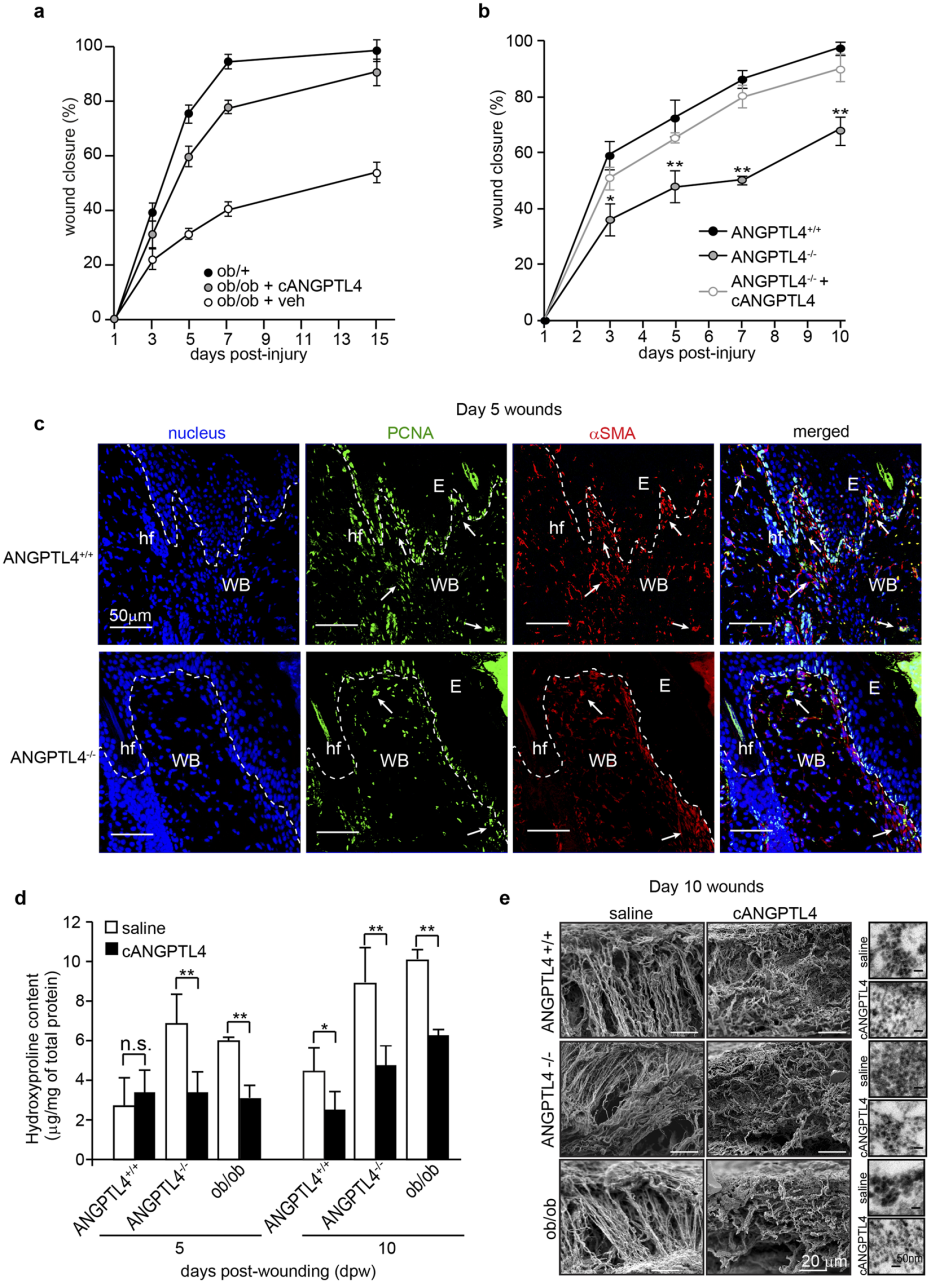
cANGPTL4 accelerates wound closure, modifies wound collagen expression and architecture, and improves the biomechanical quality of healed skin. (**a**) Wound closure rates of saline (veh) or cANGPTL4-treated ob/ob mice wounds and non-diabetic ob/+ mice wounds. Mice were inflicted with a 5 mm full-thickness excisional skin wound exposing the lower dermis. Recombinant cANGPTL4 protein or saline in 4% carboxymethylcellulose hydrogel was topically applied to the wounds (~6.25 µg/25 g body weight/day) and protected with an occlusive dressing (Tegaderm™; 3 M, USA). Wound dimensions were measured at the indicated time points before mice were sacrificed for histology and protein analysis of wounds. (**b**) Wound closure rates of saline-treated or cANGPTL4-treated wounds from ANGPTL4^+/+^, ANGPTL4^−/−^ mice. Wounds were treated with saline or cANGPTL4 and measured accordingly as stated in (**a**). (**c**) Immunofluorescence staining of day 5 wounds from ANGPTL4^+/+^ and ANGPTL4^−/−^ mice. Day 5 was chosen as a reference time point because the wound healing rate was exponential. The dotted line indicates the epidermis-dermis junction. PCNA staining in green identifies proliferating cells within the wound bed (WB). αSMA staining in red identifies wound fibroblasts. Arrows denote proliferating wound fibroblasts. E: epidermis; hf: hair follicle; scale bar: 50 μm. (**d**) Tissue hydroxyproline level of saline-treated and cANGPTL4-treated wounds from ANGPTL4^+/+^, ANGPTL4^−/−^and diabetic ob/ob mice. The amount of hydroxyproline was determined from a hydroxyproline standard curve and normalized against the total protein. (**e**) Representative EM images of connective tissue near the wound bed region and cross-sectional images of collagen fibril sizes (right inserts) obtained by tunneling electron microscopy of ANGPTL4^+/+^, ANGPTL4^−/−^ and ob/ob mice wounds treated with saline or cANGPTL4. Scale bar: 20 μm. Values represent mean ± SD, n = 5, *P < 0.05, **P < 0.01.

To understand the role of ANGPTL4 in collagen expression during normal and impaired wound healing, we first examined the rate of wound closure and the amount of collagen expression in diabetic (ob/ob), wild-type (ANGPTL4^+/+^) and ANGPTL4-knockout (ANGPTL4^−/−^) mice inflicted with a 5-mm full-thickness excisional skin wound. As expected, ob/ob wounds healed slowly when compared with cognate non-diabetic ob/+mice. In ob/ob mice, recombinant cANGPTL4 resulted in faster wound closure compared to saline treatment (Fig. 1a, Supplementary Fig. S1a). Consistent with the role of ANGPTL4 in wound repair, ANGPTL4^−/−^ mice exhibited delayed wound healing compared to ANGPTL4^+/+^ mice, which was rescued by the topical application of recombinant cANGPTL4 (Fig. 1b, Supplementary Fig. S1b).

Histological analysis of day 5 wounds when the wound healing rate was exponential revealed that ANGPTL4^−/−^ mice wounds contained fewer PCNA-positive keratinocytes and fibroblasts compared with ANGPTL4^+/+^ mice wounds. The number of wound fibroblasts was also reduced in ANGPTL4^−/−^ mice wounds (Fig. 1c). This suggested that impaired fibroblast function within the dermis also contribute to delayed wound closure of ANGPTL4^−/−^ mice. Dermal fibroblasts have indispensable roles during wound healing, actively migrating and proliferating into the site of injury to deposit extracellular matrix (ECM) proteins to facilitate wound closure, with the collateral consequences of scar-associated collagen production. As hydroxyproline is a major component of collagen fibrils, we examined the hydroxyproline content of ANGPTL4^+/+^, ANGPTL4^−/−^ and ob/ob mice wounds, with or without recombinant cANGPTL4 treatment. ANGPTL4^−/−^ and ob/ob mice wounds had elevated hydroxyproline content (Fig. 1d). Topical treatment with cANGPTL4 reduced hydroxyproline content in ANGPTL4^−/−^ and ob/ob wounds to levels comparable with cognate saline-treated wounds (Fig. 1d). We also noticed a lower hydroxyproline content in cANGPTL4-treated ANGPTL4^+/+^ wounds only at day 10 post injury, suggesting that exogenous recombinant cANGPTL4 protein, applied at wound site nearing wound closure when endogenous cANGPTL4 has significantly decreased, was sufficient to reduce scar-associated collagen production expression. There was little effect of exogenous recombinant cANGPTL4 on hydroxyproline content of ANGPTL4^+/+^ wounds on day 5, most likely because of the already high endogenous cANGPTL4^14^.

The collagen architecture of regenerated scar-free wounds is distinctly different from normal wound scars^5, 6^. To assess the collagen architecture in the regenerated (day 10) wounds of cANGPTL4 treated and non-treated ANGPTL4^+/+^, ANGPTL4^−/−^ and ob/ob mice, we performed scanning and transmission electron microscopy (Fig. 1e). The collagen fibrils of saline-treated wild-type ANGPTL4^+/+^ wounds exhibited unidirectional aligned fibers reminiscent of scar formation in adult human wounds. Similar collagen architecture, albeit with thicker fibers, was observed in ANGPTL4^−/−^ and diabetic ob/ob wounds. Notably, exogenous application of recombinant cANGPTL4 resulted in thinner collagen fibers that adopted a random, basket-weave appearance (Fig. 1e).

Scar tissues have compromised biomechanical properties compared to uninjured skin. Since cANGPTL4 treatment reduced the scar-like features of regenerated mice wounds compared with vehicle treatment, we tested whether the biomechanical properties of healed mice skin were also improved by cANGPTL4 treatment. A section of healed mouse skin was carefully removed and cut into a specific geometric shape for biomechanical analysis, such that the healed segment resided at the narrowest region of the tissue (Supplementary Fig. S1c). The stress-strain relationship derived from the tensile test provides insight into the biomechanical properties of healed wounds. In the tensile test, the skin specimen was subjected to failure and the relationship of force versus extension was determined. The maximal tensile strength is the maximum strain that the wound tissue can tolerate before breaking. A high value represents low fragility of the skin tissue. Tissues from all cANGPTL4-treated healed wounds exhibited a higher maximal tensile strength than tissues from cognate saline-treated wounds (Supplementary Fig. S1d). We also calculated the toughness and Young’s modulus properties based on the stress-strain curve. Toughness equals the total area underneath the stress-strain curve. Young’s modulus determines a specimen’s tendency to deform elastically; a higher elastic modulus meant higher deformation resistance of the skin. Saline-treated wound tissues exhibited significant reduction of toughness and Young’s elastic modulus compared with both cANGPTL4-treated wound tissue and unwounded skin tissue (Supplementary Fig. S1e,f). Overall, cANGPTL4 treatment improved the biomechanical properties of the healed skin.

Our mouse experiments indicated that cANGPTL4 treatment produced a physiological endpoint with reduced scar-associated collagen deposition within wounds. We further examined the effect of recombinant cANGPTL4 on full-thickness splinted excisional wound healing and collagen expression in wild-type rats. The topical application of cANGPTL4 accelerated wound healing (Supplementary Fig. S1g,h) and Van Gieson staining of wound sections revealed reduced collagen expression at the wound bed of cANGPTL4-treated compared with saline-treated rat wounds (Supplementary Fig. S1i). This was further confirmed by lower hydroxyproline content in cANGPTL4-treated rat wounds (Supplementary Fig. S1j). Overall, our animal experiments indicated that cANGPTL4 reduces collagen expression, modifies collagen architecture and improves the biomechanical qualities of healed skin. These outcomes suggest a role for cANGPTL4 in wound healing and scar management.

### cANGPTL4 reduces scar-associated COL1A2 and COL3A1 expression

**Figure 2:**
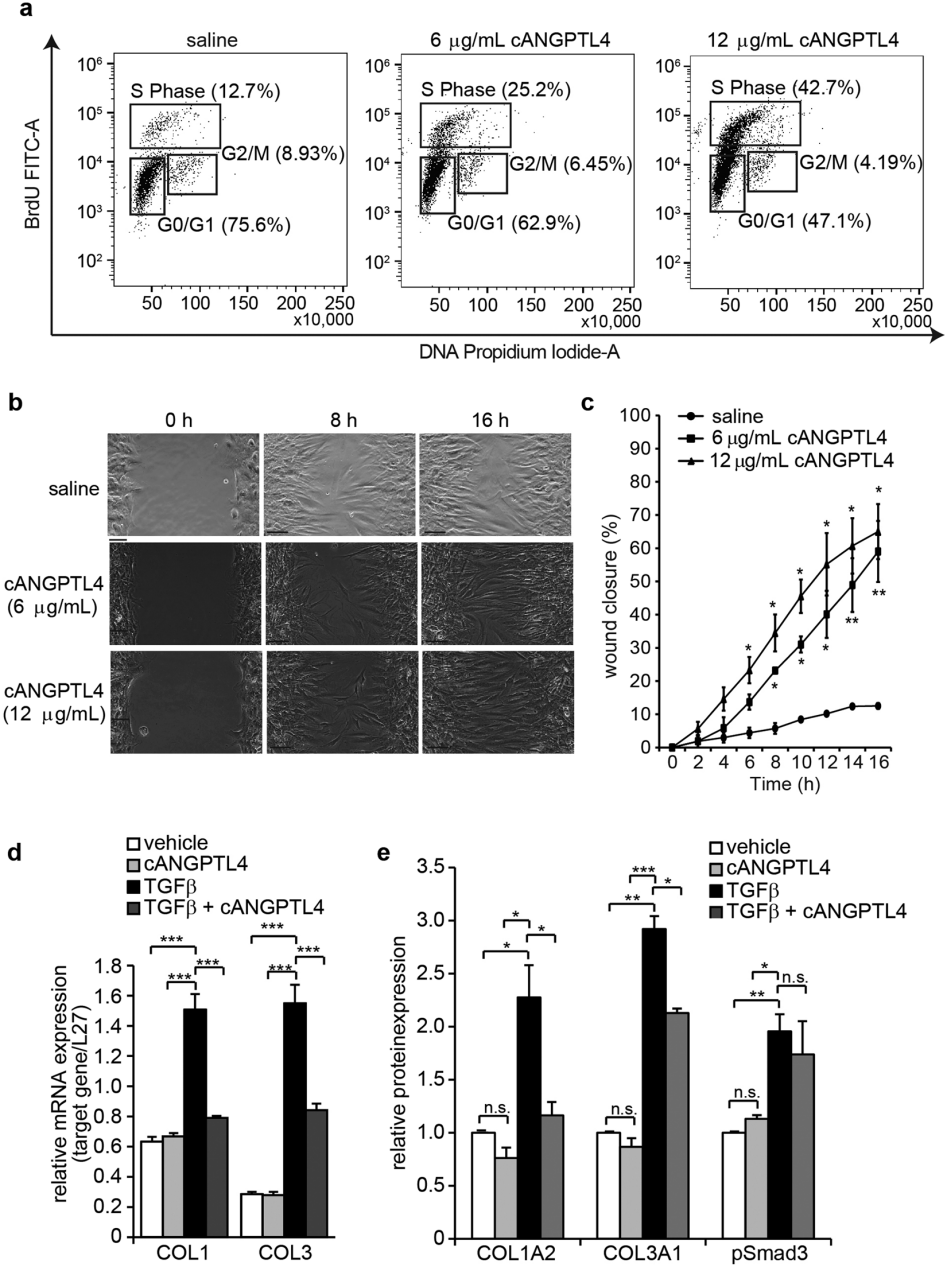
cANGPTL4 enhanced fibroblast proliferation and migration and reduced fibroblast collagen expression. (**a**) Representative FACs scatter plots of fibroblasts subjected to 24 h of the indicated treatments, then pulsed with 40 μM BrdU for 30 min prior to FAC S analysis. Fibroblasts were harvested and fixed in 70% ice-cold ethanol, their DNA was denatured with 1.5 M HCl, rinsed to neutralize acidity, then stained with FITC-conjugated anti-BrdU antibody and propidium iodide according to manufacturer’s protocol. Cell populations in the G0/G1, S and G2 phases are gated and the percentages are indicated in parentheses (**b,c**) Representative images and measurements of percentage wound closure by fibroblast migration. Human primary dermal fibroblasts (1 × 10^4^ cells per insert) were seeded into silicone inserts (ibidi, USA) on a 24-well culture plate (Corning Life Sciences, USA) and allowed to attach overnight in a humidified incubator with 5% CO_2_ at 37 °C before the removal of the inserts. Fibroblasts were then pre-treated with mitomycin-C (1 mg/mL of culture media) for 2 h to induce cell cycle arrest prior to the start of the scratch assay. Culture medium was replaced with fresh media containing the indicated treatments. The plate was then placed into the Cell-IQ machine for real-time imaging. Wound closure rate was assessed using the on-board analysis software that measured the percentage change in surface area occupied by fibroblasts. (**d**) Relative mRNA expression of COL1 and COL3 in fibroblasts treated for 24 h with vehicle (saline) or 12 μg/mL cANGPTL4 in the presence or absence of 10 ng/mL TGFβ. Ribosomal protein L27 was used as housekeeping gene for normalization of target gene expression. (**e**) Relative protein expression of COL1A2, COL3A1 and phospho-Smad3 in fibroblasts treated for 24 h with vehicle (saline) or 12 μg/mL cANGPTL4 in the presence or absence of 10 ng/mL TGFβ. Bars represent mean densitometry measurements for COL1A2 and COL3A1 (normalized against β-tubulin), and phospho-Smad3 (normalized against total Smad3). Loading controls were obtained from the same sample lysates. Images are representative of 5 fields of view from n = 5 independent experiments; values are means ± SD, n = 5 independent experiments. **P < 0.01 (Mann-Whitney U-test).

We observed a reduction in the number of PCNA-positive fibroblasts and an overall reduction of wound fibroblasts in ANGPTL4^−/−^ compared with ANGPTL4^+/+^ wounds (Fig. 1c), suggesting that fibroblast proliferation and migration was impaired in the absence of cANGPTL4. To confirm whether the above responses in our animal models are also relevant to humans, we examined whether cANGPTL4 had any effect on human fibroblast proliferation. We subjected saline- and cANGPTL4-treated fibroblasts to the BrdU fluorescence-activated cell sorting (FACS) assay. Our results showed that cANGPTL4 dose-dependently increased the percentage of fibroblasts that were in the S-phase of the cell cycle, indicating enhanced proliferation (Fig. 2a). Live imaging revealed a dose-dependent improvement of fibroblast migration upon cANGPTL4 treatment. Scratch wound closure approached 60% within 16 h with 12 µg/mL cANGPTL4 as compared to only 10% closure with saline treatment (Fig. 2b,c). Our present findings with fibroblasts are similar to our previous study that cANGPTL4 facilitates cell migration via integrin cycling^11^.

Despite the increased fibroblast proliferation and migration *in vivo*, the hydroxyproline content in mice wounds was reduced by cANGPTL4 treatment. To evaluate whether this was a fibroblast-specific response, we treated human fibroblasts with saline or cANGPTL4 in the presence or absence of transforming growth factor-β1 (TGFβ1) (10 ng/mL) *in vitro*, then measured their mRNA expression of scar-associated COL1A2 and COL3A1. TGFβ1 is a major inducer of fibroblast activation and concomitant collagen expression in both mice and humans. In the absence of TGFβ, cANGPTL4 treatment did not change the baseline expression of COL1A2 and COL3A1 transcripts. Expectedly, TGFβ resulted in a strong elevation of COL1A2 and COL3A1 mRNA and protein expression. Correspondingly, phospho-Smad3 protein expression was increased by TGFβ1. cANGPTL4 treatment significantly attenuated the TGFβ1-induced increase in COL1A2 and COL3A1 mRNA expression (Fig. 2d,e, Supplementary Fig. S1k). However, phospho-Smad3 expression was unchanged between the TGFβ1 and the TGFβ1 + cANGPTL4 experimental conditions. Therefore, our *in vitro* observations also suggest that fibroblast migration and proliferation is increased by cANGPTL4, while collagen production by wound fibroblasts is reduced. Since TGFβ1 is required for the upregulation of collagen transcripts and more accurately reflects the *in vivo* wound condition, subsequent experiments to assess the effects of cANGPTL4 on fibroblasts *in vitro* were performed in the presence of TGFβ1 as the cognate control.

### cANGPTL4 regulates COL1A2 and COL3A1 expression via a bHLH-dependent signaling mechanism

**Figure 3:**
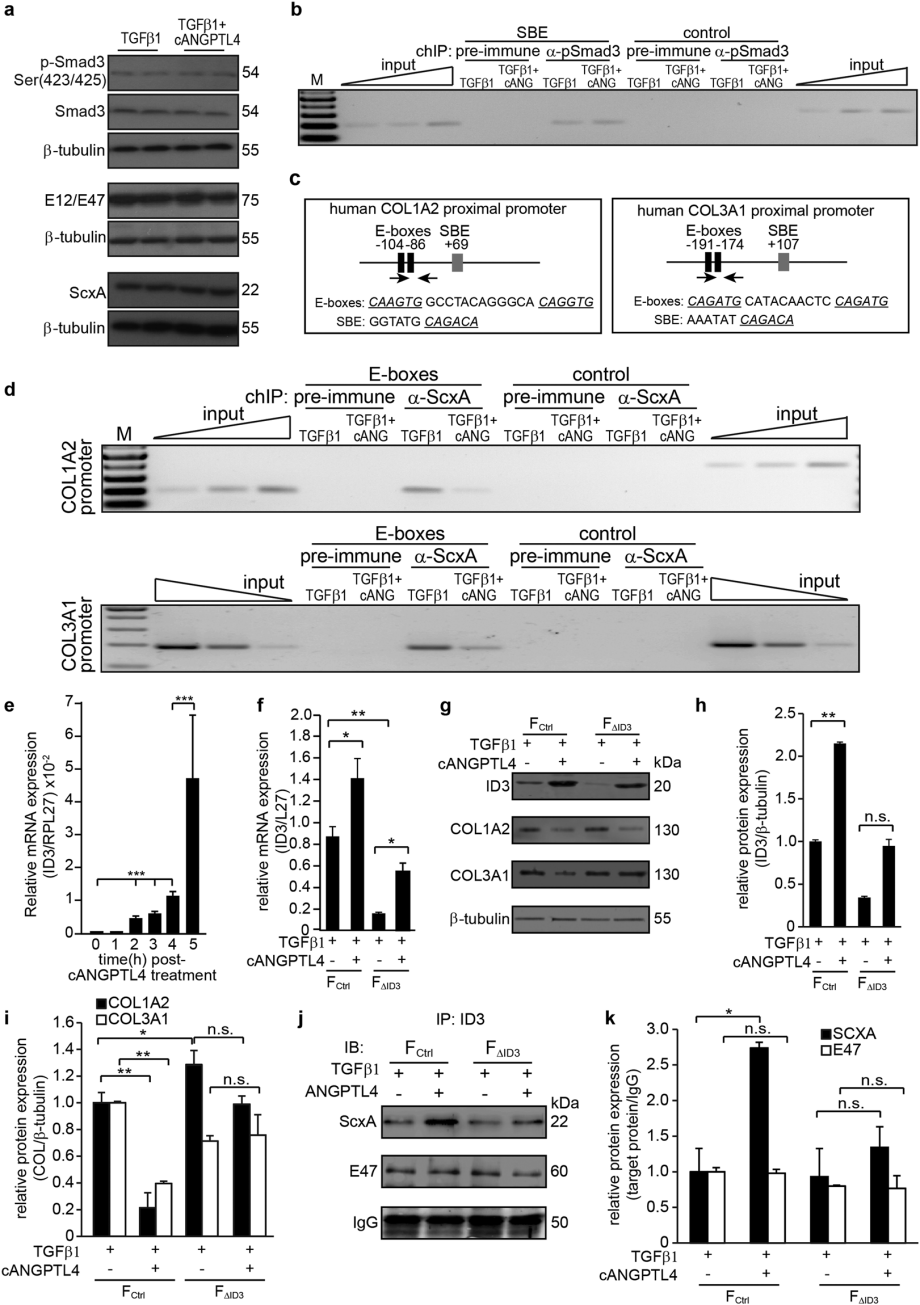
cANGPTL4 regulates COL1A2 and COL3A1 expression in fibroblasts via a bHLH-dependent signaling mechanism. (**a**) Immunoblot analysis of indicated proteins from fibroblasts treated with cANGPTL4 (12 µg/mL) in the presence or absence of TGFβ1 (10 ng/mL). β-tubulin, as loading control, was from the same samples. (**b,d**) Chromatin immunoprecipitation (ChIP) of SBE sites in the human COL1A2 gene promoter region and E-box sites in the human COL1A2 and COL3A1 gene promoter regions using phospho-Smad3 antibodies and scleraxis (ScxA) antibodies respectively. The gene sequences spanning the SBE and E-box sites and a random control sequence were analyzed by PCR from immunoprecipitated chromatin from fibroblasts treated with TGFβ1 in the presence or absence of cANGPTL4 (cANG). Pre-immune serum was used as a control. PCR was performed using chromatin before immunoprecipitation as input. M, 100 bp DNA ladder. (**c**) A schematic diagram showing the relative positions of Enhancer boxes (E-Boxes) and Smad binding elements (SBE) on the human COL1A2 and COL3A1 proximal promoters. Sequences of E-Boxes and SBE are shown. ChIP PCR primers designed specifically to bind scleraxis along this proximal promoter region are indicated. (**e**) Relative mRNA expression of ID3 in fibroblasts treated with 12 µg/mL of cANGPTL4 and lysed for mRNA at the indicated time points post-treatment. (**f**) Relative mRNA expression of ID3 in control (F_ctrl_) and ID3-knockdown (F_∆ID3_) fibroblasts treated for 16 h with 10 ng/mL of TGFβ1 (control) or TGFβ1 plus 12 µg/mL of cANGPTL4. (**g–i**) Immunodetection of ID3, COL1A2 and COL3A1 in human dermal fibroblasts subjected to the indicated treatments. (**j,k**) Immunodetection of ScxA and E47 in the immunoprecipitate of ID3. β-tubulin or IgG, as loading controls, were from the same samples/immunoprecipitates. Cropped images are representative of immunoblots from n = 5 independent experiments; full-length immunoblots scanned using the Li-Cor Odyssey® CLx system can be found in Supplementary Fig. S4a–g; values represent mean ± SD from n = 5 independent experiments. *P < 0.05, **P < 0.01,***P < 0.001 (Mann-Whitney U-test).

The pro-scarring consequence of TGFβ1 is effected through its canonical intracellular mediator Smad3 to regulate collagen synthesis^15–18^. To gain insight into human collagen scar formation, we first verified if cANGPTL4 affected Smad3-mediated transcription of collagen in TGFβ1-treated human primary dermal fibroblasts. We did not detect any difference in the protein expression of phospho-Smad3 in TGFβ1-stimulated fibroblasts co-treated with either saline or cANGPTL4 for 24 h (Fig. 3a). Chromatin immunoprecipitation (ChIP) showed that phospho-Smad3 bound specifically to the Smad3-binding site in the promoter of the human COL1A2 gene in TGFβ1-treated fibroblasts (Fig. 3b). Smad3 occupancy was not significantly affected in fibroblasts co-treated with cANGPTL4 (Fig. 3b). These observations suggest that cANGPTL4 reduces collagen expression without antagonizing the canonical TGFβ-Smad3 signaling in fibroblasts.

*In silico* analysis of the proximal promoter of the human COL1A2 and COL3A1 gene promoters revealed numerous E-boxes, which are specific DNA sequences recognized by basic helix-loop-helix (bHLH) transcription factors (Fig. 3c). Of interest, the bHLH transcriptional factor scleraxis (ScxA) was shown to synergize with Smad3 to regulate fibroblast collagen synthesis^19, 20^. ScxA requires the heterodimerization with E12/47 to activate transcription of downstream target genes. We observed no significant difference in the total expression level of ScxA protein and E12/47 (Fig. 3a). To determine whether cANGPTL4 affected the ScxA-mediated transactivation of the COL1A2 and COL3A1 promoters, ChIPs were performed using lysates from fibroblasts co-treated with TGFβ1 and with either saline or recombinant cANGPTL4. We detected a significant reduction in ScxA occupancy at the proximal promoter of the human COL1A2 and COL3A1 genes in fibroblasts treated with TGFβ1 + cANGPTL4 compared with TGFβ1 + saline (Fig. 3d). No amplification in the promoter of the human COL1A2 and COL3A1 genes was observed with pre-immune IgG or with a control sequence upstream of the E-boxes (Fig. 3d). These observations suggest that cANGPTL4 may stimulate the expression of an inhibitor of scleraxis.

Inhibitor of DNA-binding/differentiation proteins (IDs) comprise a family of proteins that heterodimerize with bHLH transcription factors to inhibit DNA binding of bHLH proteins like scleraxis^19, 21^. We observed an elevation of ID3 mRNA and protein expression in cANGPTL4-treated primary human fibroblasts (Fig. 3e–h). To verify if ID3 is necessary for cANGPTL4-mediated downregulation of collagen production in fibroblasts, we performed siRNA knockdown of ID3 in primary human fibroblasts and evaluated COL1A2 and COL3A1 expression in both control (F_ctrl_) and knockdown (F_∆ID3_) fibroblasts treated with saline or cANGPTL4 in the presence of TGFβ1. Knockdown efficiency was validated by qPCR and western blot (Fig. 3f–h). The protein expression of COL1A2 but not COL3A1 (molecular weights: 130 kDa) was elevated in F_∆ID3_. Notably, the effect of cANGPTL4 on COL1A2 and COL3A1 protein expression was attenuated in F_∆ID3_ compared with control (F_ctrl_) (Fig. 3g,i). To further clarify the mechanism, we performed immunoprecipitation in primary human fibroblasts using anti-ID3. Immunoblot analysis revealed that ID3 interacted with ScxA, but not with E12/E47, to attenuate their transcriptional activity (Fig. 3j,k). Immunoblot quantifications (values ± S.D.) were obtained from n = 5 independent experiments. Altogether, our observations suggest that cANGPTL4 enhanced ID3 expression to sequester ScxA and reduce the binding of ScxA to the E-boxes to activate COL1A2 and COL3A1 expression.

### cANGPTL4 upregulates ID3 via a β-catenin-dependent pathway *in vitro*

**Figure 4:**
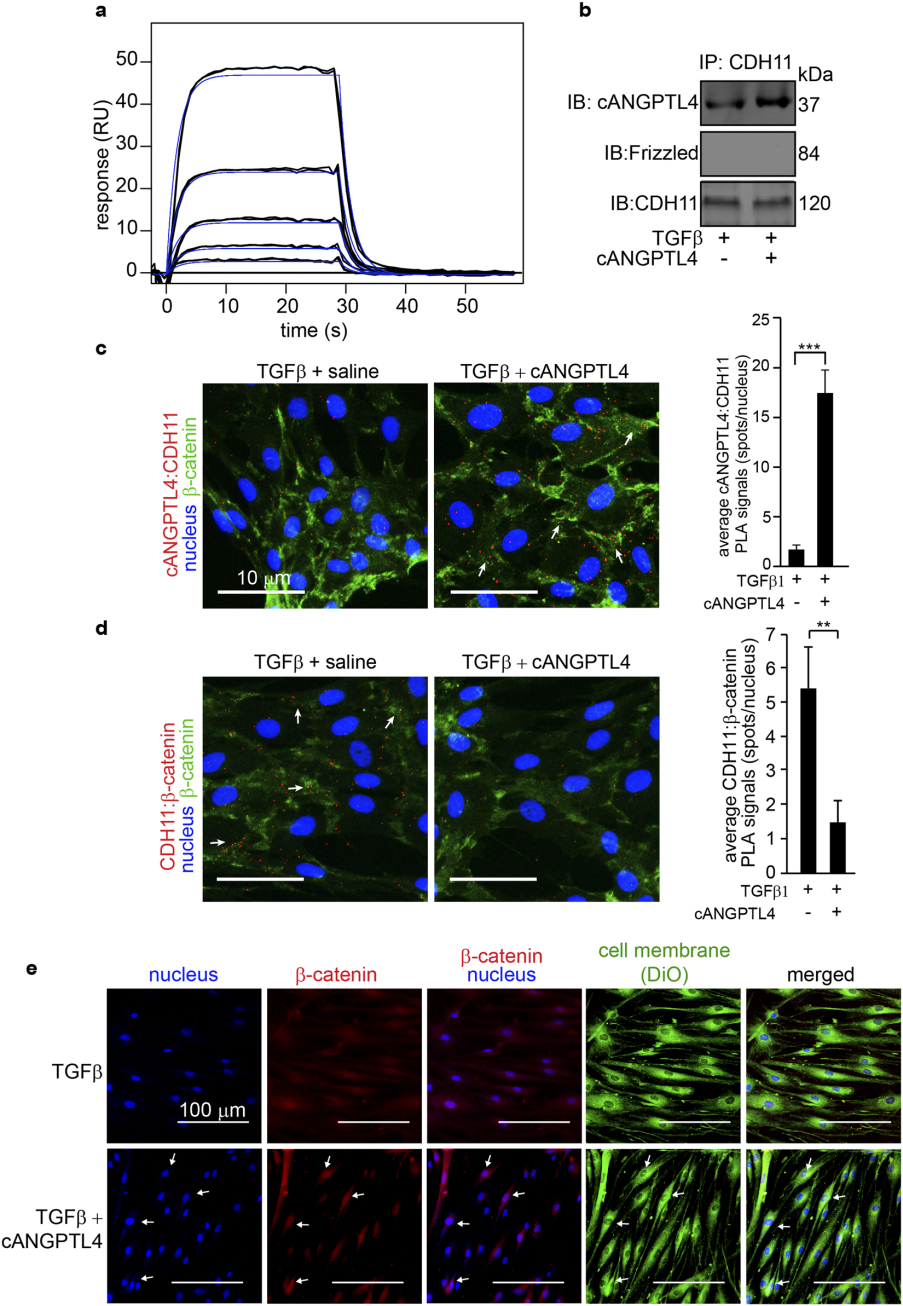
cANGPTL4 interacts with CDH11 to trigger the nuclear translocation of β-catenin in fibroblasts. (**a**) Representative sensorgram showing the interaction between recombinant cANGPTL4 and immobilized CDH11. The association and dissociation phases of the curves were analyzed using a single-site fit binding model (Langmuir 1:1 model). (**b**) Immunoprecipitation of CDH11 followed by immunodetection of indicated proteins from lysates of fibroblasts treated with TGFβ in the absence or presence of cANGPTL4. Immunoprecipitated CDH11 from the same protein samples was used as loading and transfer controls. Cropped images are representative of immunoblots from n = 5 independent experiments; full-length immunoblots scanned using the Li-Cor Odyssey® CLx system can be found in Supplementary Fig. S4h–i. (**c,d**) Detection of cANGPTL4:CDH11 (**c**) and CDH11:β-catenin (**d**) complexes in fibroblasts treated with 10 ng/mL of TGFβ1 (control) or TGFβ1 plus 12 µg/mL of cANGPTL4 by proximity ligation assay (PLA). Each PLA signal (red spot) indicates one detected interaction event. Nuclei were counterstained with DAPI (blue). Cell-cell junctions are stained in green (β-catenin). Arrows indicate co-localization of PLA signals with cell-cell junctions. Images were acquired in a single z-plane using a LSM710 META confocal laser-scanning microscope (Carl Zeiss). DAPI and PLA signals were converted to greyscale images and quantified using the Blobfinder software (Uppsala University), followed by calculation of the number of PLA spots per nucleus. Scale bar: 20 μm. Bar charts show the quantification of cANGPTL4:CDH11 and CDH11:β-catenin complexes. Values represent mean ± SD calculated from 50 nuclei per experiment condition for n = 5 independent experiments. **P < 0.01, ***P < 0.001 (Mann-Whitney U-test). (**e**) Immunofluorescence staining for β-catenin (red) in fibroblasts treated with 10 ng/mL of TGFβ1 (control) or TGFβ1 plus 12 µg/mL of cANGPTL4. Fibroblasts were counterstained with DAPI (nuclei) and VyBrant DiO dye (cell membrane). Images were acquired in a single z-plane using a LSM710 META confocal laser-scanning microscope (Carl Zeiss). Arrows indicate strong co-localization of DAPI and β-catenin signals in fibroblasts treated with 10 ng/mL of TGFβ1 plus 12 µg/mL of cANGPTL4, compared to diffuse staining in fibroblasts treated with 10 ng/mL of TGFβ1. Scale bar: 100 μm.

**Figure 5:**
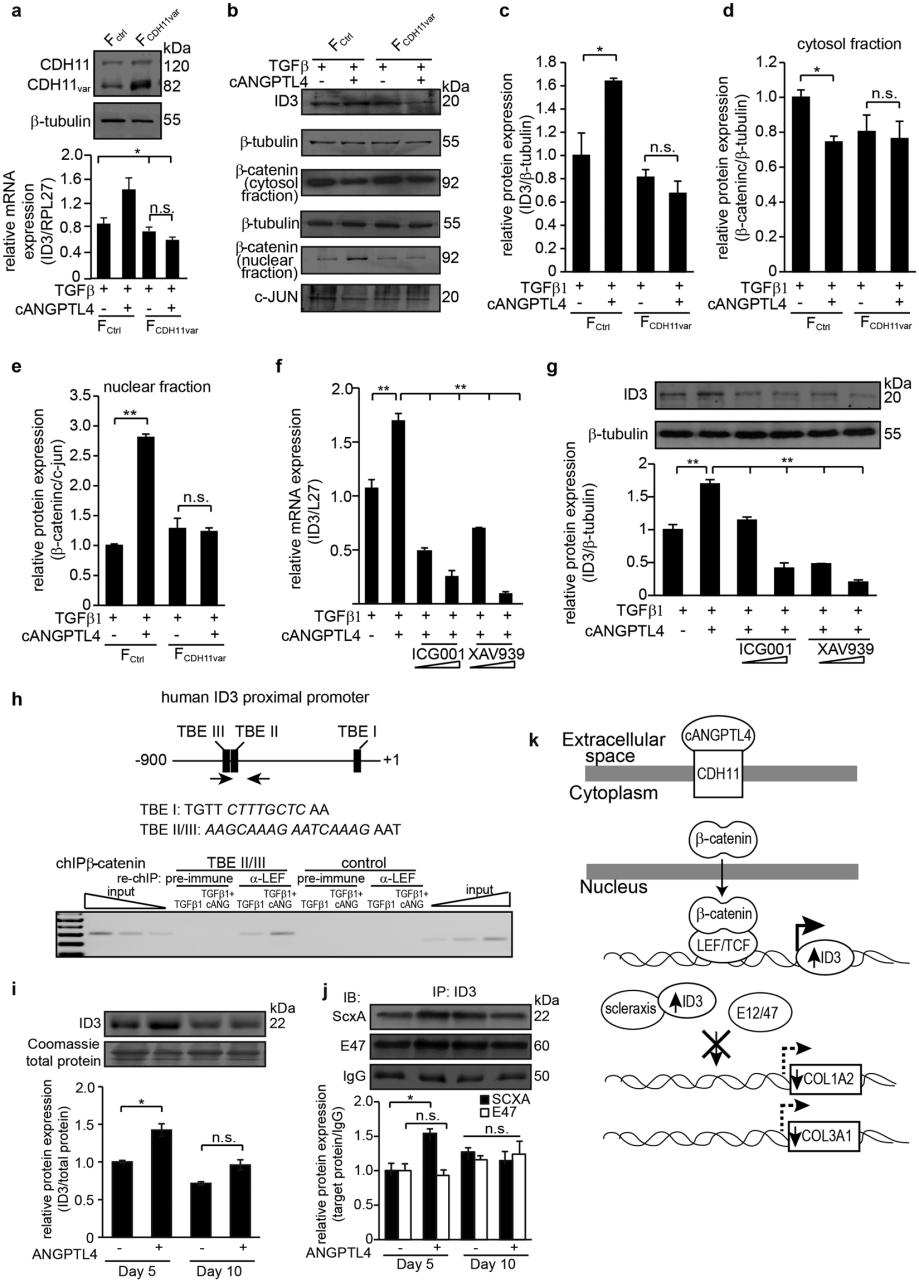
cANGPTL4 upregulates ID3 via a β-catenin-dependent pathway. (**a**) Immunoblot of full-length cadherin-11 (CDH11) and splice variant CDH11 (CDH11_var_) in human fibroblasts. Fibroblasts were transfected with empty vector (F_ctrl_) or expression vector containing cDNA encoding CDH11_var_ (F_CDH11var_). β-tubulin, as loading control, was from the same samples. Relative mRNA expression of ID3 in fibroblasts subjected to the indicated treatments. (**b–e**) Immunodetection and quantification of ID3 or β-catenin proteins in fibroblasts subjected to the indicated treatments. β-tubulin (cytosol) and c-JUN (nuclear) from the same samples were used as loading controls. Immunoblot images are cropped; full-length immunoblots can be found in Supplementary Fig. S4h–n. (**f**,**g**) Relative mRNA and protein expression of ID3 in fibroblasts treated with ICG001 and XAV939, two inhibitors of β-catenin downstream signaling. Ribosomal protein L27 was used as housekeeping control for qPCR. β-tubulin from the same samples was used as loading control for immunoblots. Values represent mean ± SD, n = 5 independent experiments. n.s., not significant, *P < 0.05, **P < 0.01, ***P < 0.001 (Mann-Whitney U-test). (**h**) ChIP of TBE on the human ID3 gene using anti-β-catenin antibody followed by re-ChIP with anti-LEF antibody. Gene sequence spanning the TBE II/III and a random control sequence were analyzed by PCR in the immunoprecipitated chromatin of fibroblasts subjected to the indicated treatments. Pre-immune serum served as control. PCR using chromatin before immunoprecipitation served as input. M, 100 bp DNA ladder. (**i,j**) Immunodetection of ID3, ScxA and E47 in the lysates and immunoprecipitates of day 5 and 10 cANGPTL4-treated wounds (n = 9). Values were normalized against total protein or ID3 anti-IgG antibodies. Loading controls were from the same samples/immunoprecipitates. Representative immunoblot images are cropped; full-length immunoblots scanned using the Li-Cor Odyssey® CLx system can be found in Supplementary Fig. S5. (**k**) Illustration depicting the mechanistic action of cANGPTL4 on COL1A2 expression in fibroblasts. cANGPLT4 interacts with fibroblast CDH11, triggering β-catenin nuclear translocation, where it interacts with TCF/LEF to transcriptionally activate ID3 gene expression. ID3 heterodimerizes with scleraxis to inhibit the transcription of COL1A2, reducing collagen secretion.

The above observations raise the question of how ANGPTL4 stimulated ID3 expression. ID3 was suggested as a transcriptional target of β-catenin-Tcf/LEF in C2C12 myoblasts, however, the Tcf/LEF binding site was not identified^22^. ANGPTL4 interacts with VE-cadherin in endothelial cells and triggers the nuclear translocation of β-catenin to stimulate gene transcription, although no target gene has been identified^23^. Emerging studies also suggested that cadherin-11 (CDH11), predominantly expressed by fibroblasts, might play important roles during wound healing, particularly by promoting fibrosis^20, 21, 24^. Thus, we hypothesized that cANGPTL4 may interact with CDH11 to trigger ID3 expression in human dermal fibroblasts during wound healing. In the first instance, we used surface plasmon resonance (SPR) to examine whether cANGPTL4 was able to directly interact with CDH11. SPR analysis revealed that cANGPTL4 interacted with CDH11with a KD value of 4.28 × 10^−8^ M (Fig. 4a). This was further corroborated by co-immunoprecipitation of cANGPTL4:CDH11 complexes (Fig. 4b). *In situ* proximity ligation assay (PLA) followed by counting of the number of PLA spots per nucleus (50 nuclei per experiment condition) revealed a significant increase in cANGPTL4:CDH11 complexes (Fig. 4c) with concomitant decrease in the number of membrane CDH11:β-catenin complexes (Fig. 4d) in TGFβ1 + cANGPTL4 fibroblasts compared to TGFβ1+ saline fibroblasts. In contrast, there was very limited interaction between cANGPTL4 and CDH2 (also known as N-cadherin) which is also expressed by fibroblasts, thereby suggesting that the cANGPTL4:CDH11 interaction dominates (Supplementary Fig. S2a). Immunofluorescence staining also revealed that there was increased co-localization of β-catenin signals with the nuclei of fibroblasts upon TGFβ + cANGPTL4 treatment as compared with cognate TGFβ + saline control (Fig. 4e), further confirmed by immunoblot for nuclear and cytoplasmic β-catenin (Fig. 5b).

As the Wnt signaling pathway is also known to regulate β-catenin^22^, we checked if the formation of cANGPTL4:CDH11 complexes engaged the Wnt receptor, Frizzled, to affect β-catenin localization. Co-immunoprecipitation with anti-Frizzled and anti-CDH11 revealed that there is no interaction between Frizzled and CDH11 while only anti-CDH11 co-immunoprecipitated cANGPTL4 (Fig. 4b, Supplementary Fig. S2b). These observations confirm that the interaction between cANGPTL4 and CDH11 triggers the translocation of membrane-bounded β-catenin to regulate ID3 expression.

To further confirm that ANGPTL4 upregulated ID3 expression through CDH11, we examined ID3 expression in fibroblasts that overexpressed a membrane-bound splice variant of CDH11 (CDH11_var_) lacking the cytoplasmic domain^25^. As previously reported, an endogenous level of CDH11_var_ was detected in fibroblasts transfected with empty expression vector^25^. Fibroblasts transfected with expression vector containing cDNA encoding for CDH11_var_ overexpressed CDH11_var_ (Fig. 5a). The overexpression of CDH11_var_ attenuated the effect of cANGPTL4 on the expression of ID3 and nuclear β-catenin, indicating that cANGPTL4 triggers CDH11-mediated β-catenin activation (Fig. 5a–e). Immunoblot quantifications (values ± S.D.) were obtained from n = 5 independent experiments. To further strengthen our observations, we examined the expression of ID3 in fibroblasts treated with two different inhibitors of the β-catenin-Tcf/LEF signaling pathway. XAV939 stimulates the degradation of β-catenin^23^, while ICG001 downregulates β-catenin-Tcf/LEF signaling by binding to CBP^26^. We observed a dose-dependent downregulation of ID3 mRNA and protein expression (Fig. 5f,g). Examination of the human ID3 promoter revealed two potential Tcf-binding sites (TBE) juxtaposed to one another (Fig. 5h). A ChIP β-catenin experiment followed by re-ChIP LEF showed that cANGPTL4 treatment increased the binding of the β-catenin:LEF complex to the TBE. No amplification was observed with pre-immune IgG or with a control sequence upstream of the TBE site on the promoter of the ID3 gene (Fig. 5h).

Having identified the mechanism of cANGPTL4-mediated suppression of collagen *in vitro*, we sought to validate our findings *in vivo* using ANGPTL4^+/+^ and ANGPTL4^−/−^ mice. Immunofluorescence staining verified co-localization of cANGPTL4 and CDH11 (Supplementary Fig. S2c). PLA analysis on day 5 wounds showed that endogenous cANGPTL4 in mice wounds binds to fibroblasts CDH11 in the dermis of ANGPTL4^+/+^ mice skin (Supplementary Fig. S2d). This interaction was largely abrogated in the ANGPTL4^−/−^ mice. Similarly, the number of cANGPTL4:CDH11 interactions was reduced in ob/ob mice wounds and rescued by exogenous recombinant cANGPTL4 application (Supplementary Fig. S2e). Correspondingly, the number of fibroblasts CDH11:β-catenin complexes was reduced in the ANGPTL4^+/+^ mice wounds as compared to those of the ANGPTL4^−/−^ mice, and cANGPTL4-treated ob/ob mice wounds displayed a reduction in CDH11:β-catenin interactions compared with vehicle controls (Supplementary Fig. S2f,g). We also detected cANGPTL4:CDH11 interactions in human scar tissues (Supplementary Fig. S3). These results indicate that the binding of cANGPTL4 to CDH11 releases β-catenin from membrane-bound complexes *in vivo* (Supplementary Fig. S2d–g). cANGPTL4-treated mice wounds also expressed higher ID3 proteins and increased interactions between ID3 and ScxA, but not with E47 (Fig. 5i,j). Immunoblot quantifications (values ± S.D.) were obtained from n = 9 mice per treatment.

Altogether, we showed that cANGPTL4 binds to CDH11, triggers the nuclear translocation of β-catenin, and stimulates β-catenin-Tcf/LEF signaling to transcriptionally upregulate the expression of ID3. Elevated ID3 interacts with the bHLH transcription factor ScxA to reduce COL1A2 and COL1A3 expression (Fig. 5k).

## Discussion

Scar formation is the physiological endpoint of skin wound repair in adults^27, 28^. It allows the rapid replacement of the lost skin tissue, although the resultant healed skin is aesthetically unattractive and has inferior biomechanical properties as compared to original unwounded skin^3, 4^. The process of scar-associated collagen production during wound healing is influenced by epithelial-stromal crosstalk. Wound keratinocytes activate autocrine signaling pathways in response to tissue damage that stimulate their migration into the wound for re-epithelialization. At the same time, keratinocyte-derived signals function in a paracrine manner on fibroblasts, recruiting them to replace the fibrin clot with collagen matrix^1, 2^. Studies have demonstrated that fibroblasts affect the amount and organization of collagen in regenerated wounds. However, the signals from the wound epithelia and the molecular mechanisms by which these signals affect fibroblast responses on scar-associated collagen production remain poorly understood.

Numerous lines of evidence have demonstrated that reduced expression of COL1A2 and COL3A1 proteins, which are essential components of fibrillar collagens type I and III respectively, results in smaller scar tissue^26, 27^. We showed that the matricellular protein, ANGPTL4, increases fibroblasts proliferation and reduces fibroblasts expression of COL1A2 and COL3A1 proteins. A similar phenotype has been observed in cultured rat skin fibroblasts treated with the synthetic PPARβ/δ agonist GW501516^29^ and in rat skin fibroblasts over-expressing the ski protein^30^. Both Ski and ANGPTL4 are direct target genes of PPARβ/δ^11, 29^. Ski acts as a transcriptional corepressor to dampen Smad3 signaling in fibroblasts. We now showed that ANGPTL4 inhibited fibroblast collagen production via a ID3-dependent mechanism triggered when ANGPTL4 binds to fibroblast CDH11. CDH11 is a classical cadherin belonging to the family of calcium-dependent cell adhesion molecules. The role of CDH11 extends beyond cell-cell adhesion as it sequesters β-catenin to the cytoplasmic face of the plasma membrane, preventing its nuclear translocation. Previous work has shown that cANGPTL4 interacts directly with the ectodomains of cadherin 5 (or VE-cadherin) on endothelial cells, declustered VE-cadherin and resulted in the nuclear translocation of β-catenin^31^. We showed a similar cANGPTL4:CDH11 interaction and a concomitant reduction of CDH11:β-catenin interactions. This was further supported by an increase in the amount of β-catenin in the nuclear fraction of cANGPTL4-treated fibroblasts. Although there is overwhelming experimental evidence showing that the loss or disruption of homophillic cadherin ectodomain interactions releases β-catenin from membrane-bound complexes^32–36^, the precise structural conformation changes in cadherin that enable the release of β-catenin have not been experimentally determined. The cANGPTL4:CDH11 interaction released membrane-bound β-catenin which entered the nucleus to trigger TCF/LEF activation of ID3 expression. ID3 heterodimerized with the bHLH transcription factor scleraxis to inhibit the transcription of COL1A2 and COL3A1, resulting in reduced collagen secretion. To our knowledge, our finding is the first to implicate a role for ANGPTL4 and scleraxis in wound healing and scar-associated collagen production. Also, most studies on scar reduction modulate the canonical TGFβ:Smad3 signaling axis. To date, no successful therapeutics for scar management have emerged from this approach, suggesting that non-canonical pathways capable of augmenting the biological readout of TGFβ are equally important. ANGPTL4 and its scleraxis-dependent mechanism may therefore prove useful when combined with pre-clinical inhibitors of canonical TGFβ signaling.

In addition to scar formation, scleraxis, a transcription factor known as a marker of tendons and ligaments, has also being implicated in other fibrotic diseases. Keloids are a dermal fibrotic disease whose etiology remains totally unknown and for which there is no successful treatment. Intriguingly, scleraxis was also induced in keloid fibroblasts, which are composed primarily of fibrillar collagens I and/or III^37^. An elevated scleraxis and reduced ski expression have been reported in renal interstitial fibrosis following obstructive kidney damage and in the healing cardiac infarct scar^38–40^. Recently, scleraxis was identified as a critical regulator of cardiac fibrotic phenotype, where an increase in scleraxis can induce collagen production. The examination of net fibrillar collagen expression in cardiac sections from wildtype and scleraxis-knockout mice revealed a clear reduction in collagen staining. Notably, collagen fibrils appeared diminished in number and size, and there was considerable reduction of perivascular collagen staining^20, 41^. Elevated expression of ID3 was also recently shown to specifically attenuate corneal fibrosis^42^. Mouse with an endothelium-specific deletion of ID3 exhibited fibrotic vasculature, cardiac enlargement and decreased cardiac function. Similar to ANGPTL4^−/−^ mice wound healing, an abnormal vascular response was also observed in the healing of excisional skin wounds of the conditional ID3-deleted mice^43^. Whether ANGPTL4 plays a role in these fibrotic diseases, such as keloids, cardiac and corneal fibrosis, infarct scar formation remains to be determined. In sum, the matricellular protein ANGPTL4 appears to be a crucial effector in normalizing the remodeling phase of wound healing by facilitating the infiltration of fibroblasts into the re-epithelializing wound while simultaneously preventing excessive collagen secretion by these fibroblasts.

Inflammation can improve or impede cutaneous repair, depending on the level and duration of inflammation, which consequently influences the time taken for wound closure and the aesthetic nature of repaired tissue^44–46^. Non-healing wounds do not proceed with the normal phases of wound repair, but remain in an inflammatory state that often leads to extended healing time, poor angiogenesis, and excessive collagen scar formation^44, 46^. Protracted inflammation is closely associated with organ fibrosis, suggesting convergent signaling pathways shared by both processes^47, 48^. The protracted increase in pro-inflammatory cytokines and recruitment of immune cells at the site of injury exacerbates fibrosis and compromises organ function. We currently lack therapeutic approaches to prevent the scarring outcome of inflammatory responses. Previously, we showed that topical application of cANGPTL4 on slow-healing diabetic mice wounds reduced infiltrating F4/80 positive macrophages and a subset of inflammatory cytokines^14^. Herein, we showed that inflamed diabetic mice wounds healed faster when treated with cANGPTL4, with reduced collagen synthesis and improved biomechanical properties of the healed tissue compared with saline-treated controls. Electron microscopy of cANGPTL4-treated collagen architecture also revealed a random basket-weave pattern reminiscent of regenerated scar-free wounds in embryos, while saline-treated wounds had adopted an orderly alignment consisting of parallel bundles of fibers typical of healed adult scar-forming wounds. These observations suggest that ANGPTL4 may also influence collagen deposition associated with immunomodulation. However, the precise roles of ANGPTL4 in the various immune cell subtypes during wound healing remain unclear. ANGPTL4 has been implicated in inflammatory response of several diseases, although the mechanism remains unknown. ANGPTL4 protects against the severe pro-inflammatory effects of saturated fat by inhibiting fatty acid uptake into mesenteric lymph node macrophages^49^. Strikingly, in ANGPTL4^−/−^ mice, saturated fat induces a severe and lethal phenotype characterized by intestinal fibrosis, fibrinopurulent peritonitis and cachexia^49^. ANGPTL4 also reduced pulmonary inflammation during influenza infection^50^. ANGPTL4 function in immune cells is best described in atherosclerosis^51, 52^. ANGPTL4 deficiency in macrophages enhances foam cell formation and M1 polarization thereby promoting plaque formation^53^. These observations suggest an immunosuppressive role for ANGPTL4. Macrophages within fibrotic tissues predominantly display the M2-phenotype. Thus, the ratio of M1:M2 polarized macrophages at wound sites will influence the extent of fibrotic response^54, 55^. Clearly, there is a need for further investigation of ANGPTL4 immuno-modulating functions and its impact on inflammation-associated fibrosis.

## Materials and Methods

### Reagents

Antibodies used: β-tubulin (H-235, sc-9104), cJUN (H-79, sc-1694) from Santa Cruz biotechnology, USA; Smad3 (ab28379), β-catenin (ab16051), β-catenin (D10A8), ANGPTL4 (ab138526) from Abcam, UK. E12/E47 (G98-271, BD Pharmingen, USA), ID3 (2B11, MA1-23242, Thermo Scientific, USA). Rabbit polyclonal CDH11 (clone 16A, MAB2014) from Merck Millipore, USA. Phospho-Smad3 (C25A9, Cell Signaling Technology, USA). ScxA (PA5-23943, Thermo Scientific, USA). COL1A2 (sc-8785, Santa Cruz, USA). COL3A1 (NB600-594, Novus Biological, USA). All IRDye conjugated secondary antibodies from LI-COR Biosciences, USA. Recombinant human TGFβ1 (Peprotech, USA) and recombinant human cANGPTL4 was produced in-house as previously described^11^.

### Cell culture conditions

Human primary fibroblasts were obtained from Asterand Bioscience in 2015 and maintained in FibroGro™-LS Complete Media Kit (Millipore, USA) in a 37 °C, 5% CO_2_, humidified incubator. Cells were used at low passage numbers (3–5 passages) and periodically tested to exclude mycoplasma contamination. 2 mM calcium was added to the culture medium to promote cell-cell junction formation in fibroblasts, as required^56^.

### *In vitro* fibroblast migration assay

Human primary dermal fibroblasts (1 × 10^4^ cells per insert) were seeded into silicone inserts (ibidi, USA) on a 24-well culture plate (Corning Life Sciences, USA) and allowed to attach overnight in a humidified incubator with 5% CO_2_ at 37 °C before the removal of the inserts. Cultured medium was replaced with fresh media containing various treatments. Fibroblasts proliferation was arrested by mitomycin C (1 mg/mL of culture media), followed by an *in vitro* scratch wound assay with the following treatments: saline, 6 µg/mL cANGPTL4 or 12 µg/mL cANGPTL4. The plate was then placed into the Cell-IQ machine for real-time photography. We assessed the closing rate using Cell-IQ analyzer software (Chip-Man Technologies, Finland).

### BrdU proliferation assay

Human primary dermal fibroblast cells (ATCC, USA) were sub-cultured at a density of 1 × 10^3^ cells/cm^2^ in 2 mL DMEM containing 1% FBS. A BrdU incorporation assay was carried out as per manufacturer’s protocol (BD Pharmingen, USA). Flow cytometry was performed on a BD Accuri C6 flow cytometer. Flowjo v10 cytometric analytical software was used for FACs analysis.

### RNA extraction and reverse transcription

Total RNA was extracted using TRIzol® Reagent followed by the PureLink™ Micro-to-Midi Total RNA Purification System according to the manufacturer’s protocol (Invitrogen, USA). Total RNA was quantified based on the A260/A280 absorbance using the Nanodrop ND1000 (Thermo scientific, USA). Total RNA was reversed transcribed using iScript Reverse Transcription Supermix for RT-PCR (Bio-Rad, USA).

### Surface plasmon resonance (SPR)

SPR was conducted using the BIACore 3000 system (BIAcore, Uppsala, Sweden) as previously described^12^. Extracellular domain of human CDH11 (Abnova) was immobilized on the CM5 chip (GEHealthcare, USA) via amine coupling (BIAcore, Uppsala, Sweden). Ethanolamine hydrochloride was injected to block any remaining ester groups. PBS was used as the running buffer. Five concentrations of recombinant cANGPTL4 protein (28.6 nM, 14.3 nM, 7.15 nM, 3.58 nM, and 1.79 nM) were injected into the CDH11 chip surfaces at a flow rate of 10 μL/min. The association (K_on_), dissociation (K_off_), and affinity (K_D_) constants were determined using Scrubber 2 software (BioLogic Software Pte Ltd). Each sensorgram was corrected by subtracting a sensorgram obtained from a reference flow cell that lacked immobilized protein.

### Proximity ligation assay

PLA experiments were performed as described by manufacturer (Olink Biosciences, Sweden) using pairs of antibodies to probe for the protein interactions^57^. Rabbit polyclonal CDH11 (1:50) was paired with mouse monoclonal anti-cANGPTL4 (1:50) or anti-β-catenin (1:50). The Duolink^®^ *In Situ* Detection Reagents Orange kit was used to reveal the interacting complexes. Sections were counterstained and mounted in Vectorshield^®^ mounting media with DAPI (Vector Laboratories, USA). Negative controls did not include the primary antibody. PLA images were taken using the LSM 710 confocal microscope (Carl Zeiss, Germany) with the Plan-Apochromat 40×/1.4 oil differential interference contrast objective. Analyses were performed with ZEN 2012 Light Edition software (Carl Zeiss, Germany) and PLA signals per nucleus (50 nuclei per experiment condition) were quantified using Blobfinder software (Centre for Image Analysis, Uppsala University).

### Chromatin immunoprecipitation(ChIP) and re-ChIP

CHIP and re-ChIP were performed as previously described^11, 58^. All primer pairs used are presented in Supplementary Table S1.

### Animals

Eight-week-old male diabetic BKS.Cg-DOCK7^m^+/+Lepr^db^/J (ob/ob) mice were purchased from The Jackson Laboratory (Bar Harbor, ME, USA). Eight-week-old male wild-type (ANGPTL4^+/+^) and ANGPTL4-knockout mice (ANGPTL4^−/−^) were obtained as previously described^50^. Eight-week-old male Sprague Dawley rats were purchased from In Vivos Pte Ltd (Singapore). All rodents used in this study were individually caged, housed in a temperature-controlled room (23 °C) on a 12-h light–dark cycle, and allowed ad libitum access to standard chow and water.

### Wounding experiments

The Institutional Animal Care and Use Committee of Nanyang Technological University approved all animal studies (ARF-SBS/NIE-A0250). Full-thickness 5 mm splinted excisional wounding of mice was performed as previously described^59^. For rats, the excisional wound diameter was 1.8 cm. Recombinant cANGPTL4 protein or saline in 4% carboxymethylcellulose hydrogel was topically applied to the wounds (~6.25 µg/25 g body weight/day) and protected with an occlusive dressing (Tegaderm™; 3 M, USA). All methods were carried out in accordance with the approved guidelines.

### Immunoblotting

Wound biopsies were homogenized with ice-cold M-PER Mammalian Protein Extraction Reagent (Thermo Scientific). Total protein lysates were resolved using 10% SDS-PAGE and electrotransferred onto Immobilon-FL PVDF membrane (Merck Millipore, USA). Membranes were blocked with 1 × Odyssey Blocking buffer (LI-COR Biotechnology, USA) for 1 h at room temperature. The membrane was then incubated overnight at 4 °C with the indicated primary antibodies in 1 × Odyssey Blocking buffer containing 0.05% Tween-20. Membranes were washed thrice with TBST (50 mM TrisHCl, pH 7.6, 150 mM NaCl, 0.05% Tween-20), and incubated with appropriate IRDye^®^680- or 800-conjugated anti-IgG secondary antibodies in 1 × Odyssey Blocking buffer containing 0.05% Tween-20 and 0.01% SDS for 1 h at room temperature. Protein bands were revealed using the Odyssey^®^CLx Infrared Imaging System, and signals were quantified using ImageStudio Software (LI-COR Biotechnology, USA).

### Tissue preparation and sections

Wound biopsies were fixed in 4% paraformaldehyde-PBS (PFA) overnight at 4 °C. Tissues were either embedded in tissue freezing medium (Leica Microsystems, USA) to be frozen immediately with liquid nitrogen, or embedded in paraffin. Cryosections of 8-µm thickness or paraffin sections of 5-µm thickness were used for histological or immunofluorescence staining.

### Hydroxyproline assay

Hydroxyproline content in biological samples was determined as previously described^60^. Trans-4-hydroxy-L-proline (0–300 µg/mL) was included to obtain the standard curve. Total protein was used for normalization.

### Biomechanical testing

Mice wound tissues at Day 10 post-wounding was chosen specifically because it was representative of the late remodelling phase of healing. A section of skin was carefully removed and cut into a specific geometry shape as indicated in Fig. 5f. Tensile strength measurements were conducted on fresh tissue using an INSTRON universal test machine (Model 1122, INSTRON Corporation, Norwood, MA), as previously described^59^.

### Transmission electron microscopy

Skin tissue biopsies were fixed with 4% PFA, 2.5% glutaraldehyde, and 0.2% picric acid in 0.1 M sodium cacodylate buffer (pH 7.6), and incubated for two weeks at 4 °C. The fixed sample blocks were incubated in 1% OsO_4_ and 1.5% potassium hexacyanoferrate in cacodylate buffer for 1 h, then dehydrated in a graded series of ethanol concentrations, and embedded in Spurr’s resin. Semi-thin sections were stained with toluidine blue and used for orientation. Ultrathin skin sections were counterstained with 7% uranyl acetate and Reynold’s lead citrate, and then examined under a JEM-1010 electron microscope operating at 80 kV or JEM-2200FS.

### Scanning Electron Microscopy

Tissue preparation for scanning electron microscopy of collagen fibers was performed as previously described with minor modifications^59^. Wound biopsies were immersed in 0.1 N NaOH at 80 °C for 1 hour to decellularize the tissue, after which they were successively transferred into 0.1 N NaOH at room temperature for 5 min, into distilled water at room temperature for 5 min. Elastic fibers were removed from the decellularized tissue by elastase treatment at 37 °C for 30 minutes at a concentration of 9.5 U/mL in 100 mM Tris buffer (pH 7.4) containing 1 mM CaCl_2_ and 0.02% NaN_3_. The decellularised tissue was then fixed in 2% glutaraldehyde and 1% paraformaldehyde overnight at 4 °C. Following fixation, the tissues were washed twice with PBS and postfixed in 1% O_s_O_4_ for 1 h. The specimens were then dehydrated in graded concentration of ethanol ranging from 50% to 100% and dried in a critical-point drying apparatus using liquid CO_2_ as immersion medium. The samples were then mounted onto a metal stub with double-sided carbon tape. After this, the specimens were sputter-coated with a thin layer of gold using SPI-Module Sputter Coater System (Structure Probe) and examined with a JSM-5410LV scanning electron microscope (JEOL).

### Statistical analysis

Statistical analyses were performed using the two-tailed Mann-Whitney U-test using SPSS Statistics (IBM Corporation, USA). *P* value < 0.05 was considered significant.

## Electronic supplementary material

Supplementary Information
