# Supplementary material for: Angiopoietin-like 4 induces a β-catenin-mediated upregulation of ID3 in fibroblasts to reduce scar collagen expression
Source: Sci Rep. 2017 Jul 24;7:6303. doi: 10.1038/s41598-017-05869-x (PMC5524754; doi:10.1038/s41598-017-05869-x)
Supplement: Supplementary file 1 — Supplementary Information [file 41598_2017_5869_MOESM1_ESM.doc]

**Angiopoietin-like 4 induces a β-catenin-mediated upregulation of ID3 in fibroblasts**

**to reduce scar collagen expression.**

Ziqiang Teo^1#^, Jeremy Soon Kiat Chan^1#^, Han Chung Chong^1*^, Ming Keat Sng^1^, Chee Chong Choo^1^, Glendon Zhi Ming Phua^1^, Daniel Jin Rong Teo^1^, Pengcheng Zhu^1^, Cleo Choong^2^, Marcus Thien Chong Wong^4^ and Nguan Soon Tan^1,3,5,6^

^1^School of Biological Sciences, Nanyang Technological University, 60 Nanyang Drive, Singapore 637551.

^2^School of Materials Science and Engineering, Nanyang Technological University, Nanyang Avenue, Singapore 639798.

^3^Lee Kong Chian School of Medicine, Experimental Medicine Building, 59 Nanyang Drive, Singapore 636921

^4^Tan Tock Seng Hospital, 11 Jalan Tan Tock Seng, Singapore 308433.

^5^Institute of Molecular and Cell Biology, 61 Biopolis Drive, Proteos, A*STAR, Singapore 138673.

^6^KK Research Centre, KK Women’s and Children’s Hospital, 100 Bukit Timah Road, Singapore 229899.

Running title: ANGPTL4 reduces collagen scars

#These authors contributed equally to the work.

*Present address: Denova Sciences Pte. Ltd., Singapore.

To whom correspondence should be address: Dr Ziqiang Teo ([zqteo@ntu.edu.sg](mailto:zqteo@ntu.edu.sg)); Mr Jeremy Soon Kiat Chan ([CHAN0693@e.ntu.edu.sg](mailto:CHAN0693@e.ntu.edu.sg)). A/P Nguan Soon Tan (senior corresponding author), School of Biological Sciences, Nanyang Technological University, 60 Nanyang Drive, Singapore 637551. Phone (+65) 6316 2941; fax (+65) 6791 3856; email [nstan@ntu.edu.sg](mailto:nstan@ntu.edu.sg).

**Keywords:** scar-associated collagen, angiopoietin-like 4, scleraxis, wound healing

**SUPPLEMENTARY INFORMATION**

**Supplementary Figures and Legends:**

**Supplementary Figure S1.** cANGPTL4 accelerated wound healing in mice and rats, improved the biomechanical properties of healed mice wounds, reduced scarring in healed rat wounds, and collagen and phospho-Smad3 expression in treated fibroblasts.

**Supplementary Figure S2.** cANGPTL4 interacts with CDH11 without engaging Wnt signaling to release β-catenin from CDH11:β-catenin membrane complexes.

**Supplementary Figure S3.** Masson’s Trichrome Stain and PLA assay for cANGPTL4:CDH11 interaction in human scar tissue.

**Supplementary Figure S4-5.** Full-length immunoblots scanned using the Li-Cor Odyssey® CLx system.

**Supplementary Table:**

**Table S1:** List of primer pairs sequences for ChIP.

**Supplementary Figures and Legends**

**
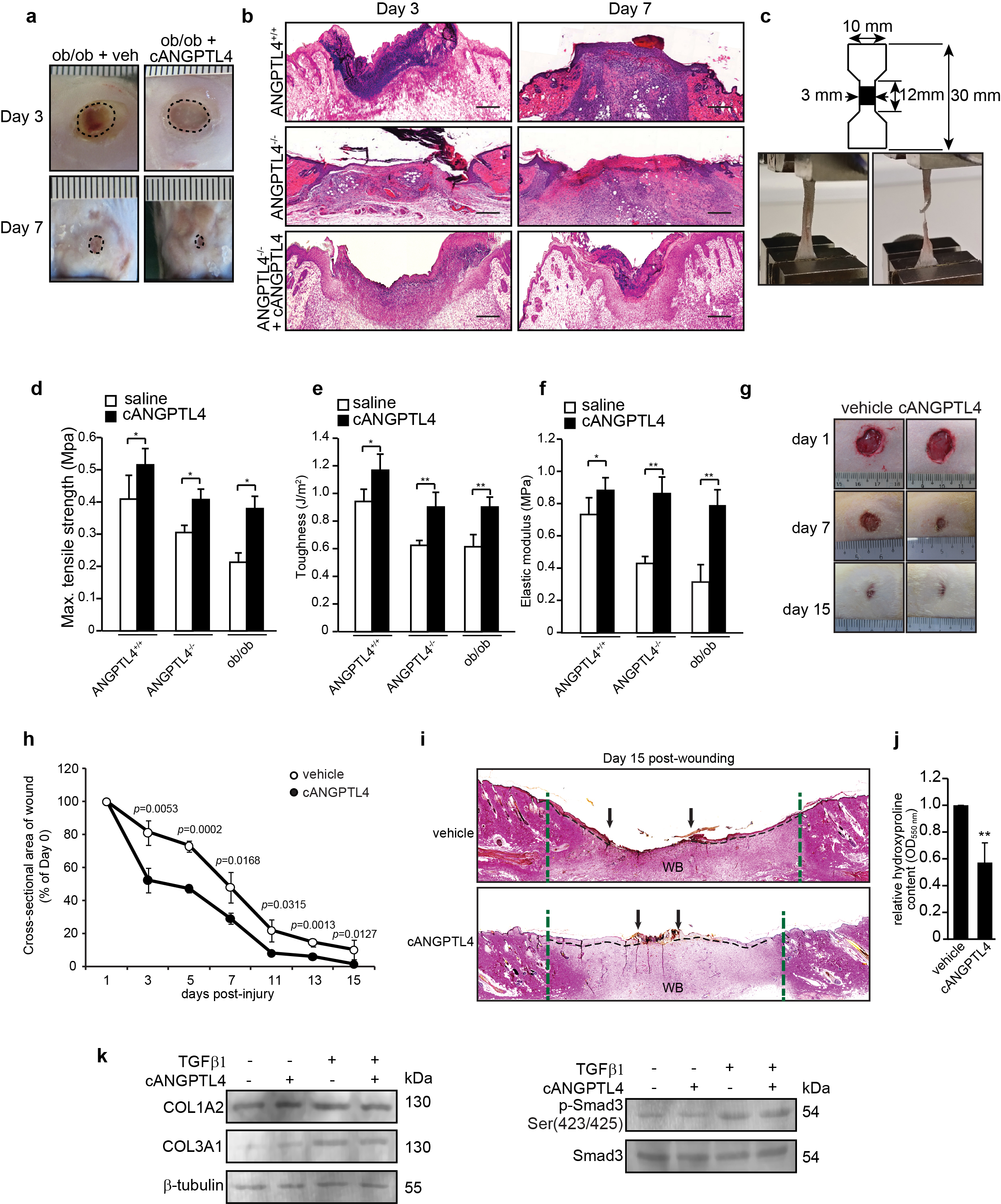
**

**Supplementary Figure S1. cANGPTL4 accelerated wound healing in mice and rats, improved the biomechanical properties of healed mice wounds and reduced scarring in healed rat wounds.** (**A**) Representative macroscopic wound images of ob/ob mice wounds treated with saline (veh) or 12 μg/mL cANGPTL4 at day 3 and day 7 post-wounding. (**B**) Representative H&E images of ANGPTL4^+/+^, ANGPTL4^-/-^ and ANGPTL4^-/-^ + cANGPTL4 wounds at day 3 and day 7 post-wounding. Scale bar: 100 μm. (**C**) A schematic diagram illustrating the geometry and dimensions of the skin wound biopsies (upper panel) and representative tensile strength measurement conducted on fresh tissue using the INSTRON test machine (lower panel). Maximum tensile strength (**D**), tissue toughness (**E**), and elastic modulus (**F**) of ANGPTL4^+/+^, ANGPTL4^-/-^ and ob/ob mice skin wounds treated with saline or cANGPTL4, derived from a stress-strain curve. Values represent mean ± SD, n = 9 per treatment. n.s., not significant, *P< 0.05 and **P<0.01 (Mann-Whitney U-test). (**G**) Representative macroscopic rat wound images at day 3 and day 7 post-wounding treated with saline (vehicle) or 12 μg/mL cANGPTL4. (**H**) Cross-sectional wound area measurements of rat wounds treated with (vehicle) or 12 μg/mL cANGPTL4. (**I**) Representative Van Gieson stain images of saline (vehicle) or 12 μg/mL cANGPTL4 treated rat wounds at day 15 post-wounding and (**J**) their respective hydroxyproline levels. Experiments with each sample were conducted in triplicate, at minimum. The amount of hydroxyproline was determined using a hydroxyproline standard curve and normalized against the total protein concentration. (**K**) Representative immunoblots for COL1A2, COL3A1 and phospho-Smad3 protein expression. β-tubulin from the same samples was used as loading and transfer control for COL1A2 and COL3A1 immunoblots. Total Smad3 from the same samples was used as loading and transfer control for phospho-Smad3 immunoblots. Values represent mean ± SD, n = 5, *P<0.05, **P<0.01.


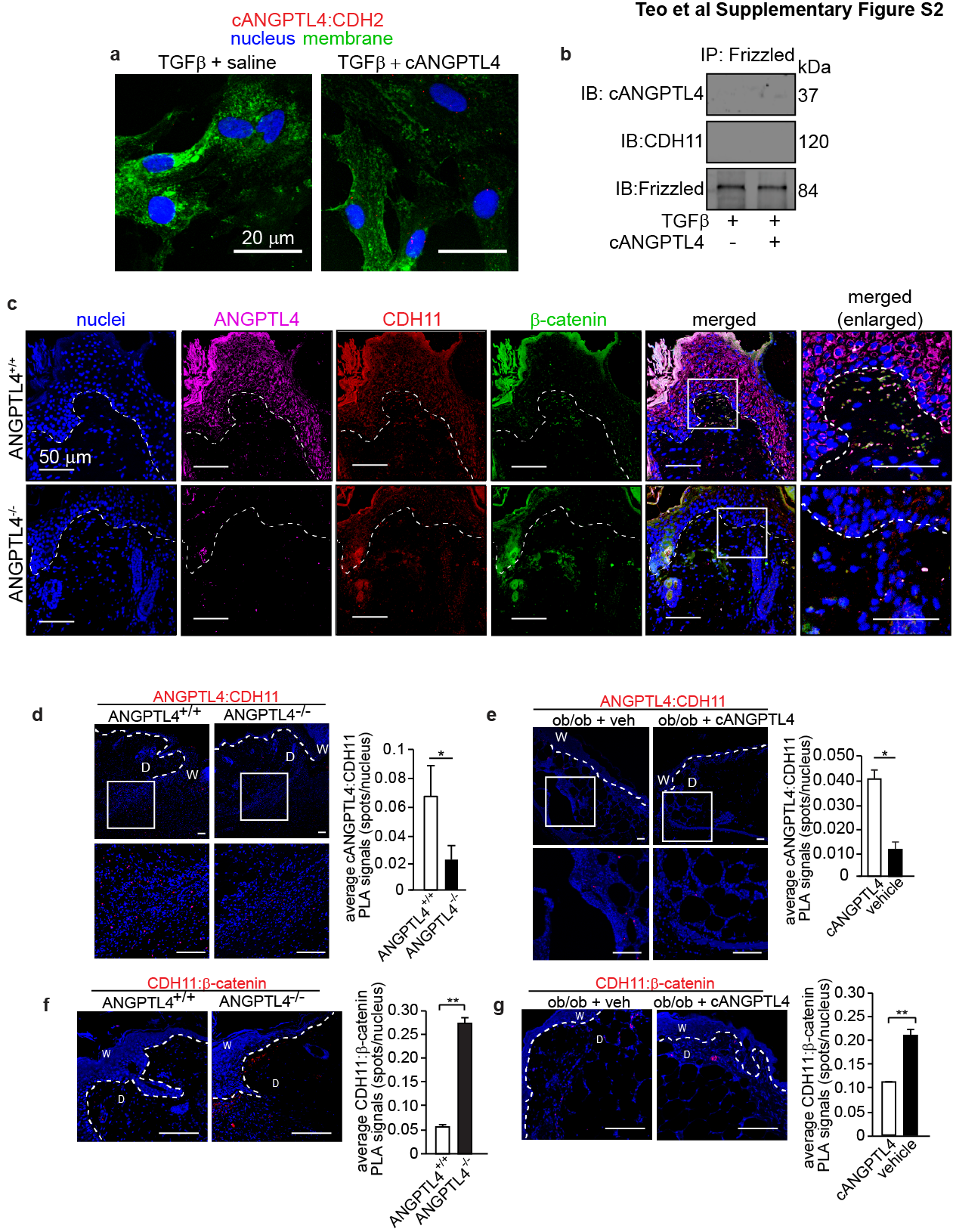


**Supplementary Figure S2. cANGPTL4 interacts with CDH11 without engaging Wnt signaling to release β-catenin from CDH11:β-catenin membrane complexes.** (**A**) Detection of cANGPTL4:CDH11 and CDH11:β-catenin complexes in fibroblasts treated with 10 ng/mL of TGFβ1 (control) or TGFβ1 plus 12 µg/mL of cANGPTL4 by proximity ligation assay (PLA). Each PLA signal (red) indicates one detected interaction event. Nuclei were counterstained with DAPI (blue). Scale bar: 20 μm. (**B**) Immunoprecipitation of Frizzled followed by immunodetection of indicated proteins from wound lysates. (**C**) Immunofluorescence staining of cANGPTL4 (magenta), CDH11 (red) and β-catenin (green) on day 5 ANGPTL4^+/+^ and ANGPTL4^-/-^ mice wounds. Arrows indicate co-localization of cANGPTL4 and CDH11 staining. E: epidermis; WB: wound bed; dotted line: epidermis-dermis junction; scale bar: 50 μm. (**D, E**) Detection and quantification of cANGPTL4:CDH11 interactions by proximity ligation assay (PLA) in ANGPTL4^+/+^, ANGPTL4^-/-^ and ob/ob mice wounds treated with with saline (veh) or 12 μg/mL cANGPTL4. Lower panels are enlarged from the regions demarcated by a white square in the above panels. (**F, G**) Detection and quantification of CDH11:β-catenin interactions by proximity ligation assay (PLA) in ANGPTL4^+/+^, ANGPTL4^-/-^ and ob/ob mice wounds treated with with saline (veh) or 12 μg/mL cANGPTL4 Dotted line: epidermis-dermis junction. D: dermis; W: wound; scale bar: 50 µm. Each PLA signal (red) indicates one detected interaction event. Nuclei were counterstained with DAPI (blue). Graphs show mean ± SD for number of PLA signals (ANGPTL4:CDH11 complex) per nucleus, n = 5, *P<0.05, **P<0.01.

**
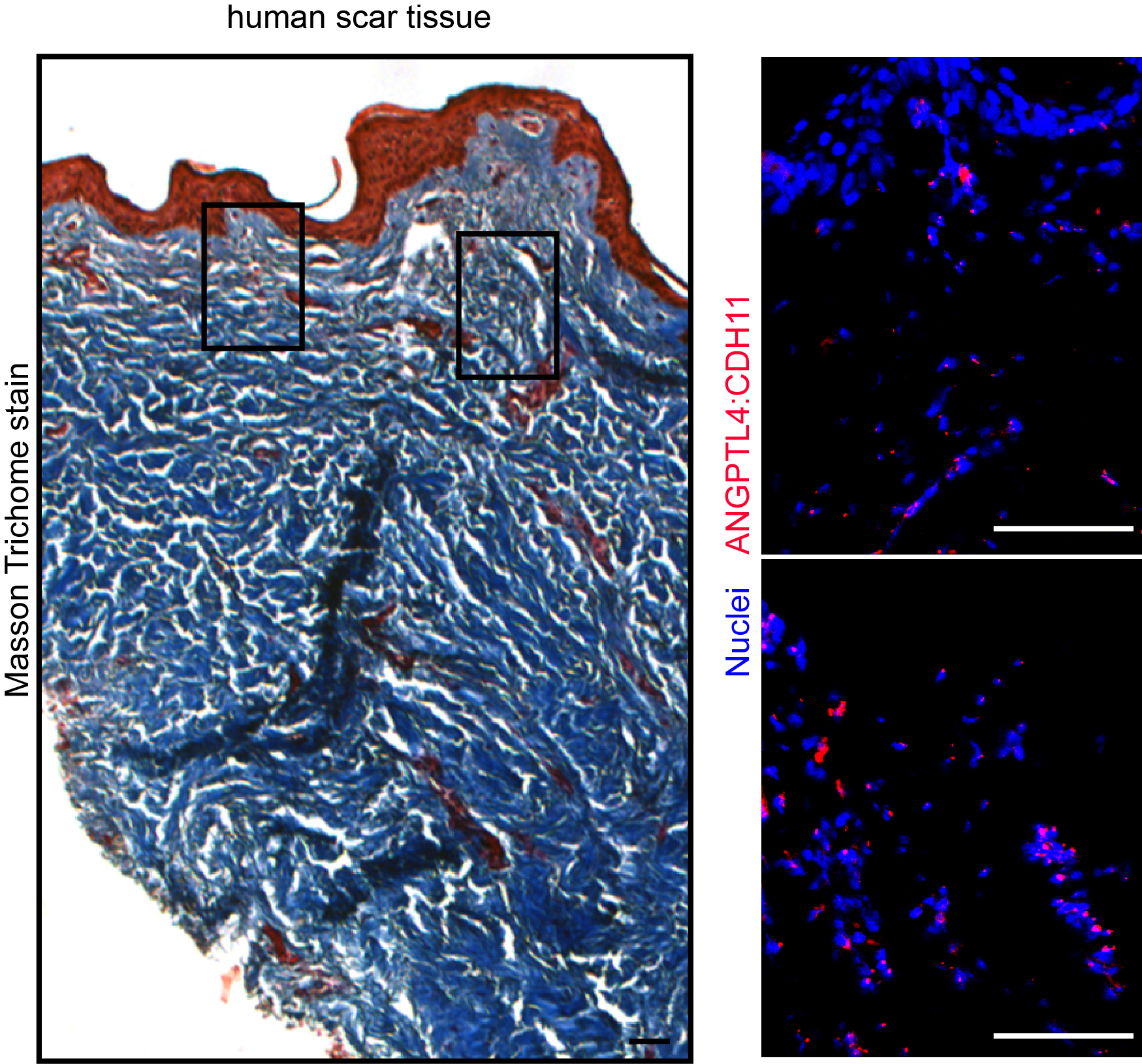
**

**Supplementary Figure S3. Masson’s Trichrome Stain and PLA assay for cANGPTL4:CDH11 interaction in human scar tissue.** Masson’s Trichrome Stain (left) of human scar tissue. Collagen is stained blue, cytoplasm is stained pink/red and nuclei are stained black. Regions highlighted by black boxes are the representative fields of view shown in the PLA assay (right). Each red spot represents one cANGPTL4:CDH11 interaction. Nuclei were counterstained with DAPI (blue). Scale bar: 100 μm.

**
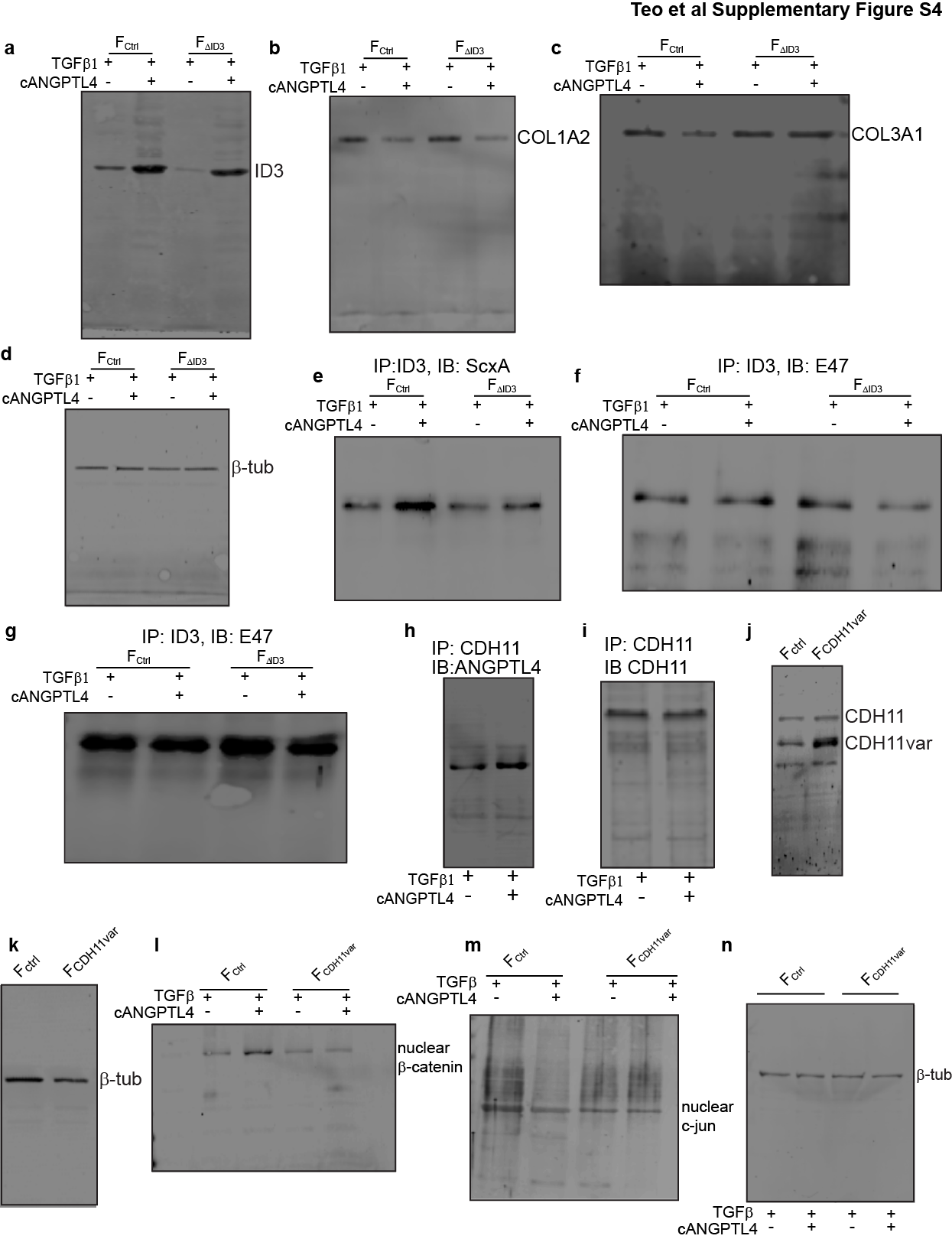
**

**Supplementary Figure S4. Full-length immunoblots scanned using the Li-Cor Odyssey® CLx system.** Infrared images were converted to greyscale using the onboard Image Studio™ software and exported as high quality .tiff files. (**A-G**) Full-length immunoblots relating to Figure 3 are shown. (**H-I**) Full-length immunoblots relating to Figure 4 are shown. (**J-N**) Full-length immunoblots relating to Figure 5 are shown.

**
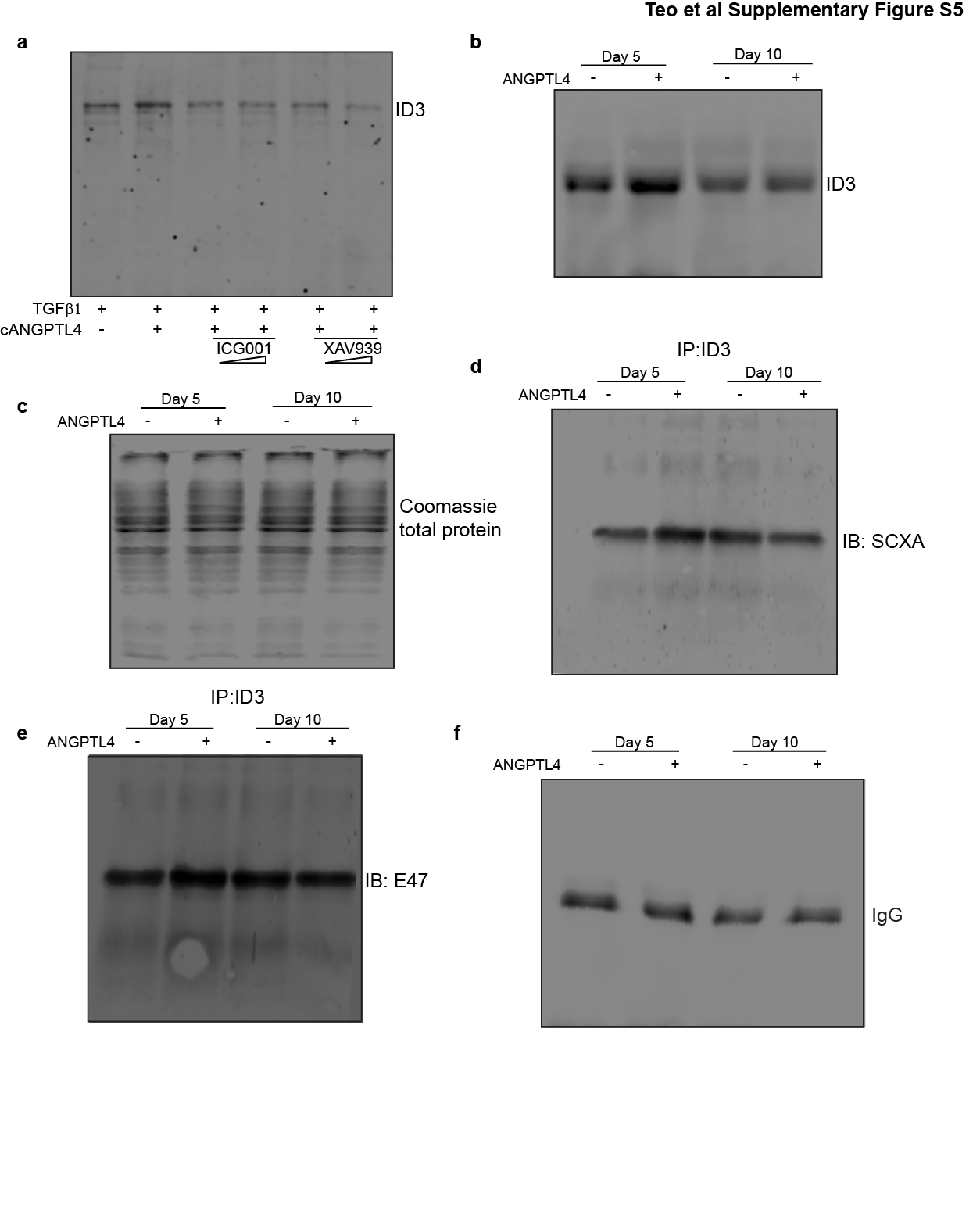
**

**Supplementary Figure S5. Full-length immunoblots scanned using the Li-Cor Odyssey® CLx system.** Infrared images were converted to greyscale using the onboard Image Studio™ software and exported as high quality .tiff files. (**A-F**) Full-length immunoblots relating to Figure 5 are shown.

**Table S1: List of primer pairs sequences for ChIP**.

| Primers for ChIP | Forward Sequence (5’ to 3’) | Reverse Sequence (5’ to 3’) |
| --- | --- | --- |
| Smad binding Site (SBE) for COL1A2 | CGGAGATCTGCAAATTCTGCC | GCTGGCTTCTTAAATTGGTTCC |
| E-boxes for COL1A2 promoter | GCCACGCTATCGAGTCTTCC | CGCCCTTTCCAAGTTTGG |
| Control for COL1A2 promoter | CTAGGCAACATGGTGAAAC | TCAAAGGTAAACATCGGAC |
| E-boxes for COL3A1 promoter | AAAACAAGGCAGAGCATTTCT | CCATCCCCTCAGCAGTAAAA |
| Control for COL3A1 promoter | TCACACTATTCAGGAATGAG | AGACTATTAATCATTGAAAG |
| TBE II/III | TCCTGTGTTAATCTTGTCTCC | AGTAGCTCCAGCTCCATCTG |
| Control for ID3 promoter | GGTGGAAAGAAAGGGAATAG | TTCACACAGGGATGCGTG |
